# Abnormal accumulation of p53 predicts radioresistance leading to poor survival in patients with endometrial carcinoma

**DOI:** 10.3892/ol.2019.10940

**Published:** 2019-09-30

**Authors:** Azusa Akiyama, Takeo Minaguchi, Kaoru Fujieda, Yoshihiko Hosokawa, Keiko Nishida, Ayumi Shikama, Nobutaka Tasaka, Manabu Sakurai, Hiroyuki Ochi, Toyomi Satoh

**Affiliations:** Department of Obstetrics and Gynecology, Faculty of Medicine, University of Tsukuba, Tsukuba, Ibaraki 305-8575, Japan

**Keywords:** p53, protein, mutation, survival, endometrial carcinoma

## Abstract

Type II endometrial carcinoma mainly originates from p53 aberration. However, the detailed prognostic significance of p53 aberration in endometrial carcinoma remains to be clarified. In the present study, abnormal p53 accumulation was analyzed using immunohistochemical techniques in endometrial carcinoma samples derived from 221 consecutive patients. The expression levels of p53 were associated with clinicopathological parameters and patient survival. P53 overexpression was observed in 37/221 patients (17%), and was associated with non-endometrioid histology, post-menopause and advanced tumor stage (III/IV; P=0.0006, P=0.03 and P=0.025, respectively). Survival analysis indicated that patients with p53-overexpressing tumors exhibited poor overall survival (OS) compared with patients without p53 overexpression (P<0.000001). Univariate and multivariate analyses demonstrated that the parameters p53 overexpression, age ≥70, non-endometrioid histology and advanced stage were significant and independent prognostic factors for poor OS (P=0.00012, P=0.00048, P=0.0027 and P=0.0015, respectively). Additionally, adjuvant radiotherapy was associated with increased OS in patients without p53 overexpression. This finding was not observed for patients with adjuvant chemotherapy. In contrast to patients without p53 overexpression, patients with p53 overexpression exhibited no association with OS (P=0.02 vs. P=0.40). Notably, adjuvant radiotherapy was identified to be a significant prognostic factor for favorable OS in the subset of patients that did not exhibit p53 overexpression and received post-operative treatment (P=0.026). The findings suggested that abnormal p53 accumulation may influence patient survival via unfavorable biological tumor properties, including rapid progression and radioresistance. The present study offered valuable insights for the genome-directed management of endometrial carcinoma.

## Introduction

Endometrial carcinoma is the most common malignancy of female genital organs in developed countries, and the incidence is recently increasing (1). The standard primary treatment is composed of surgery with or without postoperative chemotherapy and/or radiotherapy based on stratification by the risks for recurrence. Endometrial carcinoma is conventionally categorized into two major classes, namely type I and II. Type I tumors are generally characterized by endometrioid histology, precancerous atypical hyperplasia, perimenopausal incidence, obesity, superficial myometrial invasion, favorable prognosis, and frequent *PTEN* mutations (2,3). Type II tumors are generally characterized by non-endometrioid histology, *precancerous intraepithelial carcinoma arising* in atrophic endometrium, older age, postmenopausal status, reduced weight, deep myometrial invasion, poor disease prognosis, and frequent *TP53* mutations. The tumor suppressor protein p53 functions as the ‘guardian of the genome’ by inducing cell cycle arrest, senescence, and apoptosis in response to oncogene activation, DNA damage, and other stress signals. Loss of p53 function occurs in the majority of human tumors by mutation of *TP53* or by inactivation of the p53 signal transduction pathway. The majority of the mutations result in the expression of a p53 protein that has lost wild-type functions and exerts a dominant-negative regulation over the remaining wild-type p53 proteins. However, it has recently become apparent that mutant p53 further acquires oncogenic functions different to those resulting from loss of wild-type function (4). The majority of the mutant p53 proteins acquire oncogenic properties, such as invasion, metastasis, increased proliferation, and cell survival. Recently, a number of molecular agents targeting mutant p53 have been developed (5–9), and the efficacies for various types of malignancy are currently being examined in clinical trials. However, the precise prognostic significance of p53 aberration in endometrial carcinoma remains to be clarified. In the present study, we investigated the impact of the abnormal accumulation of p53 in tumors on the outcome of patients with the disease. The findings provide novel and useful implications for genome-directed individualized management of endometrial carcinoma.

## Materials and methods

### 

#### Patients and specimens

The Ethics Committee of the University of Tsukuba Hospital approved the study protocol. All patients diagnosed with endometrial carcinoma, who received surgery in the Department of Obstetrics and Gynecology at the University of Tsukuba Hospital between 1999 and 2009, were identified by our database. A total of 221 consecutive patients were included in the present study, and their medical records were retrospectively reviewed. The median follow-up duration was 132 months (range, 3–209 months). The follow-up data were retrieved until 2018-7-20. All samples were obtained with opt-out procedure in accordance with the study protocol approved by the Ethics Committee of the University of Tsukuba Hospital. Staging was performed based on the criteria of the International Federation of Gynecology and Obstetrics (FIGO, 2008) (10). Endometrioid carcinomas were subclassified into three grades (G_1_, G_2_, and G_3_) according to the FIGO criteria. The treatment of the patients was performed as described previously (3). Table I summarizes the patient characteristics.

#### Immunohistochemistry

**Figure 1.**
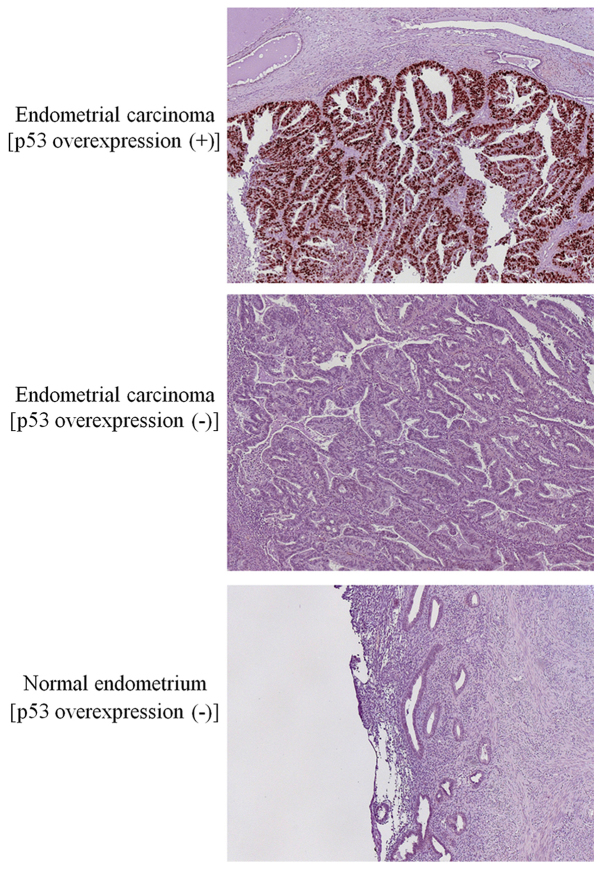
Representative immunostaining images for p53 in endometrial carcinoma and normal endometria samples. Magnification ×100.

Immunohistochemistry was performed as described previously (11). The antibodies used were the following: Anti-human p53 (DO-7) (mouse monoclonal, 1:200; Dako) and anti-human PTEN (6H2.1) (mouse monoclonal, 1:100; Cascade). The corresponding normal endometrial or stromal tissues were used as an internal positive control. The negative control samples comprised samples incubated in the absence of primary antibody that indicated low background staining. Representative immunostaining images for p53 in endometrial carcinomas and normal endometria are shown in Fig. 1.

#### Immunohistochemical (IHC) scoring

P53 and PTEN expression levels were evaluated as previously described (3,11). Briefly for p53 expression, positive staining of ≥10% of tumor cells was considered overexpression (+), and negative or positive staining of <10% of tumor cells was overexpression (−). The average value from the scores of two independent observers (AA and TM) blinded to the clinicopathological variables was used as the final value. Normal endometrial samples from 15 women were used as control samples, and 100% of the specimens were negative for p53, whereas more than 90% exhibited a score value of 6 for PTEN expression.

#### Statistical analysis

The differences in the proportions were evaluated by the Fisher's exact test. Kaplan-Meier survival curves were calculated and compared using the log-rank test. The Cox proportional hazard model was used for the univariate and multivariate analyses.

## Results

IHC analysis demonstrated p53 overexpression in 37 out of 221 patients (17%). P53 overexpression was significantly associated with non-endometrioid histology, non-G1, post-menopause, and advanced FIGO stage (III/IV) (P=0.0006, 0.004, 0.03, and 0.025, respectively, Table II).

**Figure 2.**
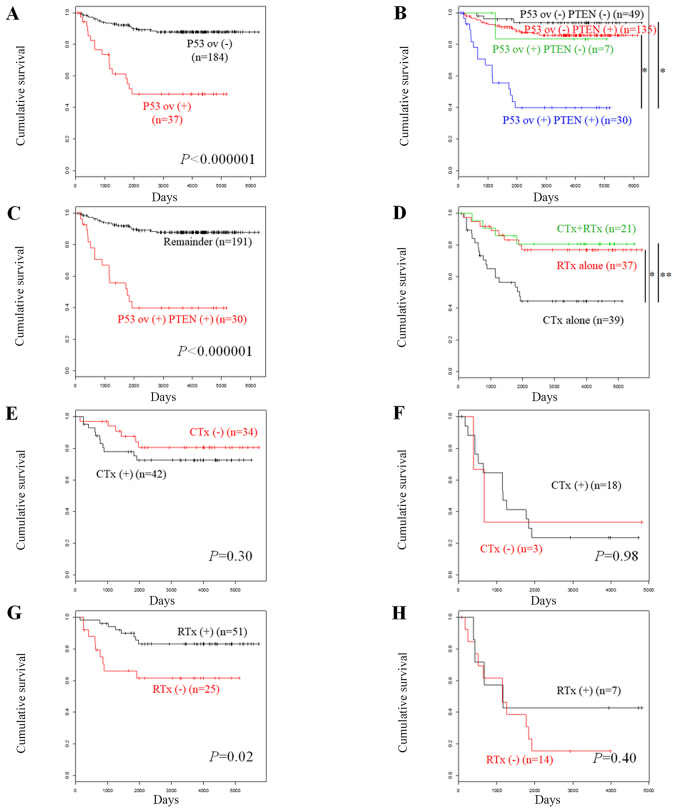
Kaplan-Meier curves were constructed in order to assess overall survival according to protein expression levels in endometrial carcinoma. (A) Patients without p53 overexpression (n=184) vs. those with p53 overexpression (n=37). (B) Patients with no p53 overexpression and negative PTEN (n=49), no p53 overexpression and positive PTEN (n=135), p53 overexpression and negative PTEN (n=7), and p53 overexpression and positive PTEN (n=30). *P<0.000001, as indicated. (C) Patients with p53 overexpression and positive PTEN (n=30) vs. the remaining subjects (n=191). (D) Patients who received adjuvant chemotherapy alone (n=39), adjuvant radiotherapy alone (n=37) and both therapies (n=21). *P=0.004 and **P=0.01, as indicated. (E) Patients without p53 overexpression, who received adjuvant chemotherapy (n=42) vs. those who did not receive adjuvant chemotherapy (n=34). (F) Patients with p53 overexpression, who received adjuvant chemotherapy (n=18) vs. those who did not receive adjuvant chemotherapy (n=3). (G) Patients without p53 overexpression, who received adjuvant radiotherapy (n=51) vs. those who did not receive adjuvant radiotherapy (n=25). (H) Patients with p53 overexpression, who received adjuvant radiotherapy (n=7) vs. those who did not receive adjuvant radiotherapy (n=14). CTx, chemotherapy; ov, overexpression; RTx, radiotherapy.

Survival analysis demonstrated that patients with p53-overexpressing tumors exhibited significantly poor overall survival (OS) compared with the patients who did not exhibit p53 overexpression (Fig. 2A, P<0.000001). Univariate analysis for unfavorable prognostic factors indicated that the parameters p53 overexpression, age higher than and/or equal to 70 years (≥70), non-endometrioid histology, advanced FIGO stage (III/IV), myometrial invasion higher than ½, and lymphovascular space invasion were significantly associated with OS (P<0.00001, <0.00001, <0.00001, <0.00001, <0.00001, and 0.00011, respectively, Table III). Subsequent multivariate analysis indicated that the parameters p53 overexpression, age ≥70, non-endometrioid histology, and advanced tumor stage were significantly associated with OS (P=0.00012, 0.00048, 0.0027, and 0.0015, Table III).

In addition, the OS was compared according to the expression levels of p53 and PTEN. Loss of PTEN expression was a prognostic indicator for favorable OS in endometrial carcinoma (3). Patients with p53 overexpression (−) and PTEN (−) tumors were associated with favorable disease prognosis, followed by those with p53 overexpression (−) and PTEN (+) tumors and those with p53 overexpression (+) and PTEN (−) tumors. The patients with p53 overexpression (+) PTEN (+) tumors exhibited unfavorable prognosis (Fig. 2B). Patients with p53 overexpression (+) PTEN (+) tumors exhibited significantly lower OS compared with that noted in the remaining patients (P<0.000001, Fig. 2C).

We further compared OS according to the modalities of adjuvant therapies in patients who received post-operative treatment. Patients who received adjuvant chemotherapy alone indicated significantly lower OS compared with that noted in patients with adjuvant radiotherapy alone or with both adjuvant therapies (Fig. 2D, P=0.004 and 0.01, respectively). The effects of the adjuvant therapies on the disease prognosis were dependent on the p53 status. Adjuvant chemotherapy did not influence OS in patients without p53 overexpression (Fig. 2E, P=0.30) or with p53 overexpression (Fig. 2F, P=1.0). By contrast, adjuvant radiotherapy significantly increased OS in patients without p53 overexpression (Fig. 2G, P=0.02). This effect was not noted in patients with p53 overexpression (Fig. 2H, P=0.40). We further conducted univariate analyses of the effects of the adjuvant therapies on the OS of the patients with p53 overexpression compared with those without p53 overexpression (Table IV). While adjuvant chemotherapy did not influence OS in patients with or without p53 overexpression [hazard ratio, 0.98 (95% confidence interval, 0.22–4.37) *vs*. 1.64 (0.61–4.45), Table IV], adjuvant radiotherapy increased OS in patients without p53 overexpression, but not in patients with p53 overexpression [HR, 0.34 (95% CI, 0.13–0.88) *vs*. 0.61 (0.19–1.93), Table IV]. Univariate analysis of various prognostic factors in patients without p53 overexpression who received adjuvant therapies demonstrated that with the exception of adjuvant radiotherapy being significant for improved OS (P=0.026, Table V), the parameters age ≥70, non-endometrioid histology, and advanced tumor stage were significant for unfavorable OS (P=0.010, 0.0081, and 0.019, respectively, Table IV). However, subsequent multivariate analysis indicated that only the parameter age ≥70 was a significant and independent prognostic factor for OS (P=0.039, Table V).

## Discussion

Wild-type p53 protein is susceptible to ubiquitin-mediated degradation by the proteasome, whereas mutant p53 is not, resulting in abnormal accumulation of the protein in p53-mutant tumors. The IHC analysis conducted in the present study revealed abnormal accumulation of p53 in 17% of endometrial carcinomas. This finding was in line with the previously published frequencies of *TP53* mutations in endometrial cancer (12).

In addition, the association of the IHC data with the clinicopathological parameters was examined. P53 overexpression was significantly associated with non-endometrioid histology and advanced-stage disease (Table II). Furthermore, survival analyses indicated that p53 overexpression was a significant and independent prognostic factor for poor OS (Table III). These findings suggested that tumors harboring p53 aberrations may have aggressive biological behavior, such as rapid progression. This effect may contribute to the prognostic impact of p53 with regard to the poor patient survival. We further compared OS according to the p53/PTEN expression of the patients. Previously we reported that negative PTEN expression is a prognostic indicator for favorable OS in endometrial carcinoma (3). Patients with p53 overexpression (+) PTEN (+) tumors exhibited considerably lower OS compared with that noted in the remaining patients (Fig. 2B and C), suggesting that they may be managed as the highest-risk group with the most aggressive phenotype.

The comparison of OS according to the modalities of the adjuvant therapies in the patients receiving post-operative treatment indicated that the improvement in their survival by adjuvant radiotherapy correlated with their p53 overexpression (−) status, while adjuvant chemotherapy did not improve OS irrespective of the p53 status (Fig. 2E-H, Table IV). Furthermore, univariate analysis in patients without p53 overexpression who received adjuvant therapies revealed that adjuvant radiotherapy, but not adjuvant chemotherapy, was a significant prognostic factor for improved OS (Table V). These findings suggested that the effect of p53 on poor prognosis may be partially mediated by the attenuated radiosensitivity of the tumors caused due to p53 aberration. The p53 signaling pathway is known to play critical roles in determining radiosensitivity by diverse mechanisms of actions (13). It has been reported that p53 mutations increase radioresistance in certain types of tumor cells (14–16). Moreover, p53 status is associated with the disease outcome following radiotherapy in patients with specific types of malignancy (17,18). Taken collectively, the data suggest that p53 expression may serve as a radiosensitivity biomarker for endometrial carcinoma. Although the p53 pathway is known to contribute to chemoresistance in certain types of tumors, the present study did not support this hypothesis. This may be explained by the tissue-specific induction of the p53 target genes (19,20), whereby chemosensitivity and radiosensitivity may be different depending on the type of tumor.

Accumulating mutant p53 proteins are attractive targets for molecular therapy as *TP53* is the most frequently mutated gene in human malignancies. Current strategies for targeting mutant p53 are focusing on the destabilization or inactivation of its mutant form, or the reactivation of wild-type p53 function. Destabilization of mutant p53 has been addressed mainly by targeting heat shock proteins via histone deacetylase enzymes in order to rescue MDM2-dependent degradation of mutant p53 (7,8). Disruption of mutant p53 function may be achieved by preventing its interaction with other transcription factors. For example, the molecule RETRA has been shown to inhibit the mutant p53-p73 interaction and to restore p73 function (9). A number of compounds or peptides that result in the reactivation of wild-type function in mutant p53 have also been reported. Among them, two small molecules, namely PRIMA-1 (p53 reactivation and induction of massive apoptosis) and its potent methylated analog, APR-246/PRIMA-1^MET^, have been reported to convert mutant p53 to a wild-type conformation, thereby restoring its sequence-specific DNA binding and transcriptional activation (6,21–23). PRIMA-1 or APR-246/PRIMA-1^MET^ induce apoptosis in tumors with both wild-type and mutant p53 (24–27), which may be explained by the observation that both unfolded mutant p53 and unfolded wild-type p53 are refolded by PRIMA-1 (28). These compounds further activate caspase enzymes, leading to cytochrome c release from the mitochondria (29). The activity of the compounds can be enhanced by combined administration of conventional chemotherapeutics as well as molecular targeting agents, including cisplatin, carboplatin, doxorubicin, docetaxel, and olaparib (30–32). APR-246 was the first mutant p53-restoring drug, which entered clinical trials, and exhibited optimal tolerability (5,6). Currently, two phase II studies are ongoing in recurrent high-grade serous ovarian cancer with positive p53 IHC staining. One involves the treatment of platinum-sensitive disease with combined administration of carboplatin and pegylated liposomal doxorubicin hydrochloride (PLD) (PiSARRO; NCT02098343), and the other is conducted for platinum-resistant disease with combined PLD (PiSARRO-R; NCT03268382). The findings of the present study suggested that molecular therapeutics that focus on p53-targeting may sensitize p53-overexpressing tumors to adjuvant radiotherapy. This potentially leads to the improvement of patient survival in subjects with poor prognosis. The development and clinical applications of efficacious molecular agents targeting p53 are warranted in the near future.

The present study contains specific limitations. Firstly, IHC overexpression of p53 was used as a surrogate for p53 mutation, whereas its mutations were not examined. Secondly, the present study was conducted in a single institution, and the sample size was relatively small. Further studies are required to strengthen the current findings. Finally, the retrospective study design can cause potential bias, suggesting that the results must be verified by prospective trials.

In conclusion, the present study demonstrated that p53 overexpression was associated with non-endometrioid histology, post-menopause, and advanced stage, and that patients with p53-overexpressing tumors exhibited worse OS compared with those without p53 overexpression. Univariate and multivariate analyses indicated that p53 overexpression was a significant and independent prognostic factor for poor OS. Adjuvant radiotherapy correlated with improved OS in patients without p53 overexpression compared with that noted in p53-overexpressing patients, and was found to be a significant favorable prognostic factor in patients without p53 overexpression who received post-operative treatments. The current findings provide significant applications for the genome-based individualized management of endometrial carcinoma.

## Figures and Tables

**Table I.** Patient characteristics.

Characteristic	Number, n (%), (n=221)
Median age (range)	57 (26–84)
FIGO stage	
I	144 (65)
IA	110 (50)
IB	34 (15)
II	17 (8)
III	36 (16)
IIIA	13 (6)
IIIC	23 (10)
IV	24 (11)
IVA	2 (1)
IVB	22 (10)
Histotype	
Endometrioid	196 (89)
G1	115 (52)
G2	56 (25)
G3	25 (11)
Serous	12 (5)
Adenosquamous	4 (2)
Clear cell	4 (2)
Poorly differentiated	1 (0)
Undifferentiated	1 (0)
Mixed epithelial	3 (1)
Primary treatment	
Surgery	221 (100)
Lymphadenectomy	171 (77)
Lymph node sampling	21 (10)
Lymph node not removed	29 (13)
Adjuvant chemotherapy	60 (27)
TC	55 (25)
CAP	4 (2)
Adjuvant radiotherapy	58 (26)

FIGO, International Federation of Gynecology and Obstetrics; TC, paclitaxel and carboplatin combination; CAP, cyclophosphamide, doxorubicin, and cisplatin combination.

**Table II.** Association between immunohistochemistry results and clinicopathological features.

	P53 overexpression		
		
Clinicopathological variables	(+) (n=37) (%)	(−) (n=184) (%)	P-value
Age ≥70	10 (27)	26 (14)	0.084
Post-menopause	32 (86)	125 (68)	0.028
Null parity	3 (8)	34 (18)	0.151
BMI >30	3 (8)	27 (15)	0.430
DM	6 (16)	33 (18)	>0.999
Endometrioid (vs. non-endometrioid)	26 (70)	170 (92)	<0.001
G1 (vs. Non-G1)	11 (30)	104 (57)	0.004
MI>1/2	15 (41)	66 (36)	0.581
LVI	17 (46)	67 (36)	0.353
FIGO stage III/IV	16 (43)	44 (24)	0.025

BMI, body mass index; DM, diabetes mellitus; MI, myometrial invasion; LVI, lymphovascular space invasion; FIGO, International Federation of Gynecology and Obstetrics.

**Table III.** Univariate and multivariate analyses of prognostic factors for poor overall survival.

	Univariate			Multivariate		
		
Prognostic factor	HR	95% CI	P-value	HR	95% CI	P-value
P53 overexpression (+) [vs. (−)]	5.71	3.00–10.9	<0.001	3.90	1.95–7.79	<0.001
Age ≥70 years (vs. <70 years)	5.04	2.63–9.64	<0.001	3.38	1.71–6.69	<0.001
Non-endometrioid (vs. endometrioid)	5.78	2.99–11.2	<0.001	2.93	1.45–5.91	0.003
FIGO stage III/IV (vs. I/II)	8.62	4.26–17.4	<0.001	3.75	1.66–8.47	0.001
MI >1/2 (vs. ≤1/2)	5.04	2.50–10.2	<0.001	2.18	0.95–5.00	0.067
LVI present (vs. absent)	3.75	1.92–7.34	<0.001	1.70	0.80–3.61	0.165

MI, myometrial invasion; LVI, lymphovascular space invasion; FIGO, International Federation of Gynecology and Obstetrics; HR, hazard ratio; CI, confidence interval.

**Table IV.** Univariate analysis of adjuvant therapy for overall survival in patient subsets with p53 overexpression (+) vs. (−).

Prognostic factor	Subset	HR	95% CI	P-value
Adjuvant CTx	p53 ov (+)	0.98	0.22–4.37	0.980
	p53 ov (−)	1.64	0.61–4.45	0.328
Adjuvant RTx	p53 ov (+)	0.61	0.19–1.93	0.401
	p53 ov (−)	0.34	0.13–0.88	0.026

HR, hazard ratio; CTx, chemotherapy; RTx, radiotherapy; ov, overexpression; CI, confidence interval.

**Table V.** Survival analyses in patients without p53 overexpression who received adjuvant therapies.

	Univariate			Multivariate		
		
Prognostic factor	HR	95% CI	P-value	HR	95% CI	P-value
Age ≥70 years (vs. <70 years)	3.50	1.34–9.10	0.010	2.98	1.06–8.40	0.039
Non-endometrioid (vs. endometrioid)	4.63	1.49–14.4	0.008	1.71	0.48–6.12	0.406
FIGO stage III/IV (vs. I/II)	4.44	1.27–15.5	0.019	3.52	0.92–13.5	0.065
MI >1/2 (vs. ≤1/2)	2.74	0.63–12.0	0.181	–	–	–
LVI present (vs. absent)	1.91	0.71–5.17	0.202	–	–	–
Adjuvant CTx done	1.64	0.61–4.45	0.328	–	–	–
Adjuvant RTx done	0.34	0.13–0.88	0.026	0.62	0.21–1.79	0.373

HR, hazard ratio; FIGO, International Federation of Gynecology and Obstetrics; MI, myometrial invasion; LVI, lymphovascular space invasion; CTx, chemotherapy; RTx, radiotherapy; CI, confidence interval.

## Data Availability

All data generated or analyzed during this study are included in this published article.

## References

[1] Ferlay J, Colombet M, Bray F (2018). GLOBOCAN 2018. International Agency for Research on Cancer, Lyon, France.

[2] Minaguchi T, Yoshikawa H, Oda K, Ishino T, Yasugi T, Onda T, Nakagawa S, Matsumoto K, Kawana K, Taketani Y (2001). PTEN mutation located only outside exons 5, 6, and 7 is an independent predictor of favorable survival in endometrial carcinomas. Clin Cancer Res.

[3] Akiyama-Abe A, Minaguchi T, Nakamura Y, Michikami H, Shikama A, Nakao S, Sakurai M, Ochi H, Onuki M, Matsumoto K (2013). Loss of PTEN expression is an independent predictor of favourable survival in endometrial carcinomas. Br J Cancer.

[4] Muller PA, Vousden KH (2013). P53 mutations in cancer. Nat Cell Biol.

[5] Deneberg S, Cherif H, Lazarevic V, Andersson PO, von Euler M, Juliusson G, Lehmann S (2016). An open-label phase I dose-finding study of APR-246 in hematological malignancies. Blood Cancer J.

[6] Lehmann S, Bykov VJ, Ali D, Andrén O, Cherif H, Tidefelt U, Uggla B, Yachnin J, Juliusson G, Moshfegh A (2012). Targeting p53 in vivo: A first-in-human study with p53-targeting compound APR-246 in refractory hematologic malignancies and prostate cancer. J Clin Oncol.

[7] Li D, Marchenko ND, Moll UM (2011). SAHA shows preferential cytotoxicity in mutant p53 cancer cells by destabilizing mutant p53 through inhibition of the HDAC6-Hsp90 chaperone axis. Cell Death Differ.

[8] Yan W, Liu S, Xu E, Zhang J, Zhang Y, Chen X (2013). Histone deacetylase inhibitors suppress mutant p53 transcription via histone deacetylase 8. Oncogene.

[9] Kravchenko JE, Ilyinskaya GV, Komarov PG, Agapova LS, Kochetkov DV, Strom E, Frolova EI, Kovriga I, Gudkov AV, Feinstein E, Chumakov PM (2008). Small-molecule RETRA suppresses mutant p53-bearing cancer cells through a p73-dependent salvage pathway. Proc Natl Acad Sci USA.

[10] Pecorelli S (2009). Revised FIGO staging for carcinoma of the vulva, cervix, and endometrium. Int J Gynaecol Obstet.

[11] Abe A, Minaguchi T, Ochi H, Onuki M, Okada S, Matsumoto K, Satoh T, Oki A, Yoshikawa H (2013). PIK3CA overexpression is a possible prognostic factor for favorable survival in ovarian clear cell carcinoma. Hum Pathol.

[12] Berchuck A, Boyd J (1995). Molecular basis of endometrial cancer. Cancer.

[13] Zhang J, Shen L, Sun LQ (2015). The regulation of radiosensitivity by p53 and its acetylation. Cancer Lett.

[14] Concin N, Zeillinger C, Stimpfel M, Schiebel I, Tong D, Wolff U, Reiner A, Leodolter S, Zeillinger R (2000). P53-dependent radioresistance in ovarian carcinoma cell lines. Cancer Lett.

[15] Dey S, Spring PM, Arnold S, Valentino J, Chendil D, Regine WF, Mohiuddin M, Ahmed MM (2003). Low-dose fractionated radiation potentiates the effects of Paclitaxel in wild-type and mutant p53 head and neck tumor cell lines. Clin Cancer Res.

[16] Ohnishi K, Inaba H, Yasumoto J, Yuki K, Takahashi A, Ohnishi T (2004). C-terminal peptides of p53 molecules enhance radiation-induced apoptosis in human mutant p53 cancer cells. Apoptosis.

[17] Ishikawa H, Mitsuhashi N, Sakurai H, Maebayashi K, Niibe H (2001). The effects of p53 status and human papillomavirus infection on the clinical outcome of patients with stage IIIB cervical carcinoma treated with radiation therapy alone. Cancer.

[18] Skinner HD, Sandulache VC, Ow TJ, Meyn RE, Yordy JS, Beadle BM, Fitzgerald AL, Giri U, Ang KK, Myers JN (2012). TP53 disruptive mutations lead to head and neck cancer treatment failure through inhibition of radiation-induced senescence. Clin Cancer Res.

[19] El-Deir WS (2003). The role of p53 in chemosensitivity and radiosensitivity. Oncogene.

[20] Lozano G (2016). The enigma of p53. Cold Spring Harb Symp Quant Biol.

[21] Aryee DN, Niedan S, Ban J, Schwentner R, Muehlbacher K, Kauer M, Kofler R, Kovar H (2013). Variability in functional p53 reactivation by PRIMA-1(Met)/APR-246 in ewing sarcoma. Br J Cancer.

[22] Saha MN, Jiang H, Yang Y, Reece D, Chang H (2013). PRIMA-1Met/APR-246 displays high antitumor activity in multiple myeloma by induction of p73 and Noxa. Mol Cancer Ther.

[23] Shchors K, Persson AI, Rostker F, Tihan T, Lyubynska N, Li N, Swigart LB, Berger MS, Hanahan D, Weiss WA, Evan GI (2013). Using a preclinical mouse model of high-grade astrocytoma to optimize p53 restoration therapy. Proc Natl Acad Sci USA.

[24] Ali D, Jonsson-Videsater K, Deneberg S, Bengtzén S, Nahi H, Paul C, Lehmann S (2011). APR-246 exhibits anti-leukemic activity and synergism with conventional chemotherapeutic drugs in acute myeloid leukemia cells. Eur J Haematol.

[25] Nahi H, Lehmann S, Mollgard L, Bengtzen S, Selivanova G, Wiman KG, Paul C, Merup M (2004). Effects of PRIMA-1 on chronic lymphocytic leukaemia cells with and without hemizygous p53 deletion. Br J Haematol.

[26] Nahi H, Merup M, Lehmann S, Bengtzen S, Möllgård L, Selivanova G, Wiman KG, Paul C (2006). PRIMA-1 induces apoptosis in acute myeloid leukaemia cells with p53 gene deletion. Br J Haematol.

[27] Bao W, Chen M, Zhao X, Kumar R, Spinnler C, Thullberg M, Issaeva N, Selivanova G, Strömblad S (2011). PRIMA-1Met/APR-246 induces wild-type p53-dependent suppression of malignant melanoma tumor growth in 3D culture and in vivo. Cell Cycle.

[28] Lambert JM, Gorzov P, Veprintsev DB, Söderqvist M, Segerbäck D, Bergman J, Fersht AR, Hainaut P, Wiman KG, Bykov VJ (2009). PRIMA-1 reactivates mutant p53 by covalent binding to the core domain. Cancer Cell.

[29] Shen J, Vakifahmetoglu H, Stridh H, Zhivotovsky B, Wiman KG (2008). PRIMA-1MET induces mitochondrial apoptosis through activation of caspase-2. Oncogene.

[30] Mohell N, Alfredsson J, Fransson A, Uustalu M, Byström S, Gullbo J, Hallberg A, Bykov VJ, Björklund U, Wiman KG (2015). APR-246 overcomes resistance to cisplatin and doxorubicin in ovarian cancer cells. Cell Death Dis.

[31] Synnott NC, Murray A, McGowan PM, Kiely M, Kiely PA, O'Donovan N, O'Connor DP, Gallagher WM, Crown J, Duffy MJ (2017). Mutant p53: A novel target for the treatment of patients with triple-negative breast cancer?. Int J Cancer.

[32] Deben C, Lardon F, Wouters A, Op de Beeck K, Van den Bossche J, Jacobs J, Van Der Steen N, Peeters M, Rolfo C, Deschoolmeester V, Pauwels P (2016). APR-246 (PRIMA-1(MET)) strongly synergizes with AZD2281 (olaparib) induced PARP inhibition to induce apoptosis in non-small cell lung cancer cell lines. Cancer Lett.

